# Implantation of a vascular access button in mice

**DOI:** 10.1038/s41598-025-20542-4

**Published:** 2025-10-21

**Authors:** Suguru Yamauchi, Kaitlyn Ecoff, Andrei Gurau, Kristen P. Rodgers, Yuping Mei, Franck Housseau, Yun Chen, John Michel, Omkar Dhaygude, Sakura Minamisawa, Ziying Xu, Feiyu Chen, Frank Bosmans, Andreas S. Barth, Jinny S. Ha, Takumi Iwasawa, Kazunori Kato, Miki Yamauchi, Hajime Orita, Shinji Mine, Tetsu Fukunaga, Malcolm V. Brock

**Affiliations:** 1 Department of Surgery, Johns Hopkins University School of Medicine, Baltimore, MD USA; 2 Department of Esophageal and Gastroenterological Surgery, Faculty of Medicine, Juntendo University, Tokyo, Japan; 3 International Collaborative Research Administration, Juntendo University School of Medicine, Tokyo, Japan; 4 Department of Oncology and Sidney Kimmel Comprehensive Cancer Center, Johns Hopkins University School of Medicine, Baltimore, MD USA; 5 Department of Mechanical Engineering, Johns Hopkins University, Baltimore, MD USA; 6 Molecular Physiology and Neurophysics Group, Department of Basic and Applied Medical Sciences, Faculty of Medicine and Public Health, University of Ghent, Ghent, Belgium; 7 Experimental Pharmacology Group (EFAR), Department of Pharmaceutical Sciences, Faculty of Medicine and Pharmaceutical Sciences, Vrije Universiteit Brussel, Brussels, Belgium; 8 Department of Medicine, Division of Cardiology, Johns Hopkins University School of Medicine, Baltimore, MD USA; 9 Division of Thoracic Surgery, Johns Hopkins University School of Medicine, Baltimore, MD USA; 10 Institute of Life Innovation Studies, Toyo University, Tokyo, Japan

**Keywords:** Vascular access, Vascular access button, Vascular catheter, External jugular vein, Outcomes research, Preclinical research, Cardiovascular models

## Abstract

Vascular access presents unique challenges in experimental mice due to their small size and anatomical constraints. Achieving reliable vascular access is crucial for optimizing experimental outcomes, especially in protocols requiring serial blood sampling or repeated intravascular therapy. Although vascular access buttons (VABs) offer significant advantages, their widespread adoption has been limited by technical challenges and a lack of comprehensive validation regarding their safety, feasibility, and long-term management. To address these gaps, we conducted a comprehensive evaluation of VAB implantation in mice. The technical success rate was 90.2%, and the 28-day survival rate was 80.4%. Optimal catheter insertion lengths determined by intraoperative and autopsy findings, and computed tomography were 9.5 ± 0.6 mm (20–25 g), 10.1 ± 0.8 mm (25–30 g), and 11.2 ± 0.5 mm (> 30 g), respectively. Catheter patency was analyzed by stratifying the cohort into groups based on physical parameters such as catheter tip geometry, heparin concentration in the maintenance solution, and frequency of catheter maintenance procedures. Although approximately half of the mice lost complete catheter patency by day 14, the majority maintained partial patency at day 28 in all experimental groups except for two cases of complete occlusion in the 2 Fr square tip, low-dose heparin, weekly maintenance group. The evaluation of biodistribution and clearance with indocyanine green indicated that VAB administration may have advantages over conventional venipuncture. These standardized methodologies can provide a framework for diverse biomedical research applications, enhancing both the efficiency and reproducibility of studies requiring reliable vascular access.

**Supplementary Information:**

The online version contains supplementary material available at 10.1038/s41598-025-20542-4.

## Introduction

Mice are the predominant animal model for in vivo experiments^1,2^. Vascular access, such as blood sampling and intravenous (IV) injection, is crucial for a wide range of biomedical experiments. However, due to the small size of mice, vascular access can be challenging and requires significant technical expertise^3^. Improper vascular access can considerably affect experimental results and hinder the generation of scientifically evidenced data. In addition, repeated testing and treatment, especially in peripheral vessels such as the tail vein, can lead to insufficient volume during blood sampling, incorrect IV administration, limited availability of peripheral veins due to venous fragility, and increased likelihood of undue stress in mice, possibly necessitating changes in study design.

An effective strategy to address these challenges involves the surgical placement of chronic vascular access catheters in laboratory animals. The external jugular vein (EJV) is the most common route for catheter placement in small animals^4–7^. Two options for chronic vascular access in mice are available: (i) an exterior vascular catheter and (ii) a vascular access button (VAB). An exterior vascular catheter is surgically implanted in a blood vessel, such as the jugular vein and central vein, and exteriorized somewhere on the body, usually between the shoulder blades^8^. The VAB system is an improved version of external vascular catheters, consisting of a catheter inserted into a blood vessel and an external hub (*i.e.*, button) with a distinct port that is implanted subcutaneously, through which the vessel can be accessed from the body surface. The VAB also simplifies the process of injections and blood sampling from laboratory animals and can be implanted even for mice housed in a group. A VAB also contributes to the practice of the 3R principle in laboratory animals by enhancing animal comfort, minimizing physical and mental stress, and reducing the need for repeated venipunctures^9^.

Despite the advantages of the VAB system, only a few studies have examined its application in laboratory animals, with limited reports focusing on VAB implantation in larger species, such as rabbits^10^ and ferrets^11^. A systematic review of the literature highlights a significant gap in comprehensive studies on vascular access systems in murine models, with a notable lack of rigorous investigations specifically addressing VAB implantation methodologies and outcomes^12–14^. The limited adoption of VAB technology in standard experimental protocols stems from insufficient empirical validation of technical parameters and safety profiles. Critical aspects requiring systematic investigation include optimal catheter tip positioning, long-term catheter patency rates, and evidence-based maintenance protocols—all essential determinants of successful VAB implementation.

This study aims to provide a robust scientific foundation for the broader implementation of VAB technology in murine experimental models, facilitating enhanced reproducibility and reliability in preclinical research.

## Results

**Fig. 1:**
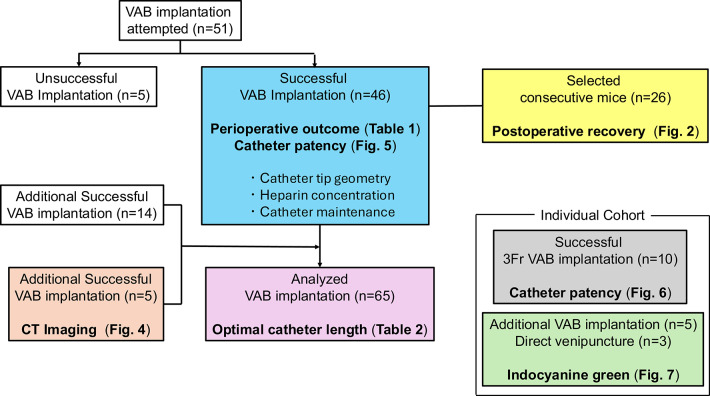
Experimental design. Flowchart for all analyses of this study. VAB, vascular access button; CT, contrast-enhanced computed tomography.

Catheter insertion into the EJV was performed using the classical cutdown method^15–18^. Supplementary Video 1 and its instructions show a step-by-step VAB implantation technique. To ensure consistency in surgical technique, all procedures in this study were performed by a single operator. If another operator was involved in a specific procedure, this was explicitly documented and taken into account during analysis. A flowchart of the analysis of all in vivo experiments performed in this study is presented as Fig. 1.

### Technical success rate

Catheter insertion into the right EJV and VAB implantation was attempted in 51 mice and the procedure was accomplished in 46 mice with a technical success rate of 90.2%. The five failed cases were due to difficulty in inserting the catheter because of the size and trajectory of the vein.

### Perioperative outcome

Thus, perioperative outcomes were analyzed in 46 mice that underwent successful VAB implantation (Table 1). The median age of the mice was 15.0 ± 5.6 weeks. The mean preoperative body weight was 26.0 ± 3.5 g. After general anesthesia, the mean operative time from disinfection of the surgical field to wound closure was 24.8 ± 5.9 min. The maximum diameter of the right EJV was measured immediately superior to the clavicle prior to catheter insertion. The mean maximum diameter was 1.73 ± 0.3 mm. Postoperative complications included bleeding (n = 4) and seroma (n = 1), but these were minor and required no additional treatment. One case of wound dehiscence (n = 1) was repaired by reoperation under general anesthesia. Complications related to catheter and VAB devices were observed on five occasions (10.8%) and included catheter dislodgement (n = 2), catheter-button disconnection (n = 2), and mechanical damage to the button (n = 1). No intraoperative mortality was observed, including in cases where the procedure was unsuccessful. The 28-day survival rate was 80.4% (37/46) among successfully implanted mice. Among the mortalities, 66.7% (6/9 mice) occurred within 7 days post-surgery. While the specific cause of death could not be determined in these cases, necropsy findings revealed no complications associated with catheter or VAB placement.

**Table 1:** Perioperative outcome.

	n = 46
Age (weeks)	15.0 ± 5.6
Sex	
Male	27 (58.7%)
Female	19 (41.3%)
Preoperative body weight (g)	26.0 ± 3.5
Surgical time (min)	24.8 ± 5.9
Intraoperative EJV diameter (mm)	1.73 ± 0.3
Postoperative complication	
Minor bleeding	4 (8.7%)
Wound seroma	1 (2.2%)
Wound dehiscence	1 (2.2%)
Catheter dislodgement	2 (4.3%)
Catheter and button disconnection	2 (4.3%)
Buttons mechanical damage	1 (2.2%)
28-day survival rate	37 (80.4%)
Within 7 days after surgery in fatal cases	6/9 (66.6%)

Values are given as the mean ± standard deviation.

### Postoperative recovery

**Fig. 2:**
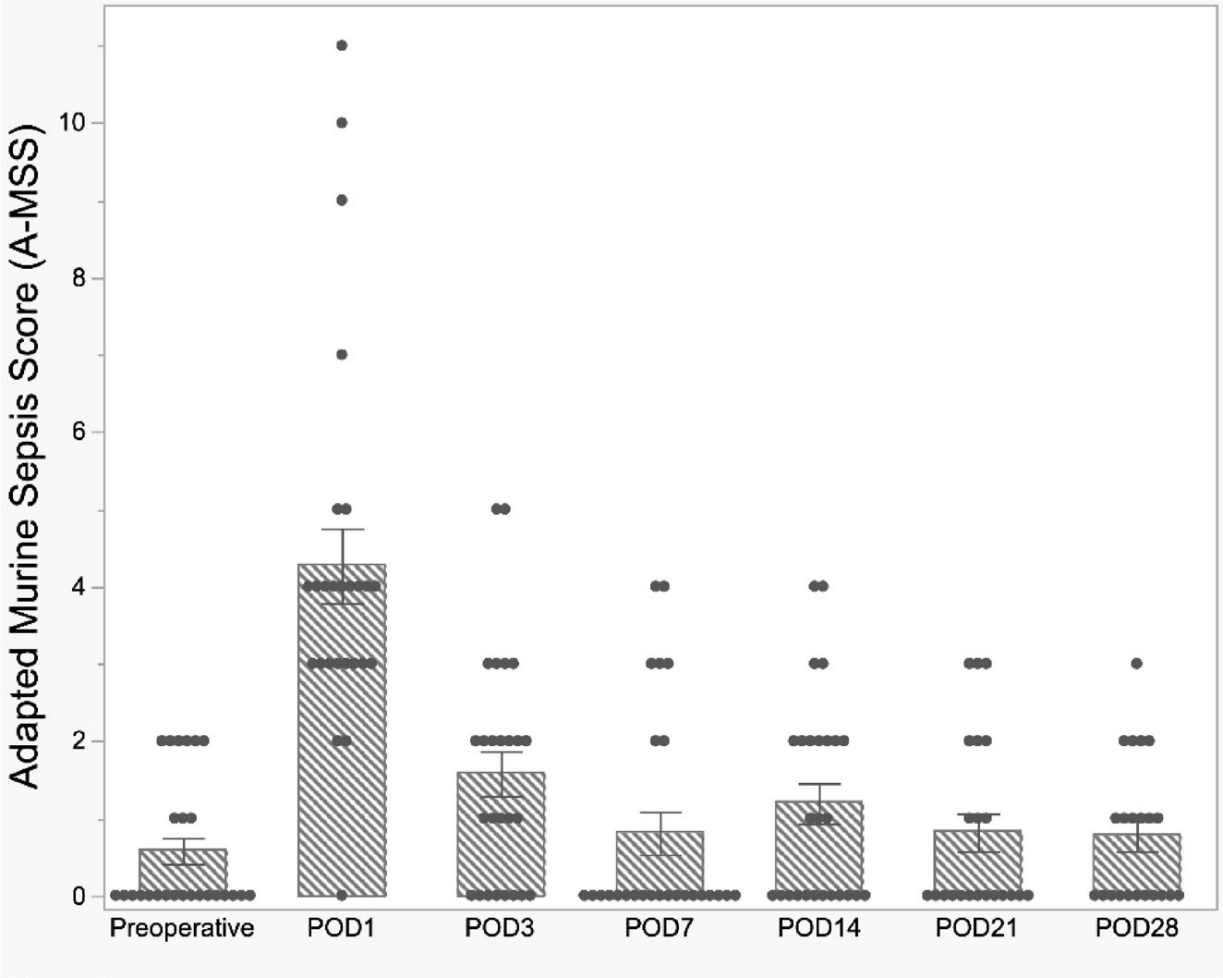
Adapted murine sepsis score (A-MSS) over the course of the postoperative period. The A-MSS score, a metric used to assess postoperative recovery in mice, typically reached its peak value on postoperative day 1 (POD1). Subsequently, the score demonstrated a gradual improvement, with most subjects returning to near-baseline values by POD7.

We analyzed the recovery of mice after surgery using changes in preoperative vs. postoperative Adapted Murine Sepsis Scores (A-MSS)^19^. A-MSS results for 26 consecutive mice, excluding cases that experienced mortalities during the postoperative observation period (Fig. 1), are shown in Fig. 2. At both postoperative day 21 (POD21) and POD28, data from four cases were lost at each time point, resulting in a final analysis of 22 cases in total. Preoperative A-MSS scores were ≤ 2 for all subjects. The highest A-MSS scores were observed on POD1, with a mean of 4.3 and a standard deviation of 2.5. A-MSS scores showed progressive improvement, returning to near-baseline levels by POD7. During the remaining follow-up period, A-MSS scores were observed to be stable.

### Catheter insertion length

**Fig. 3:**
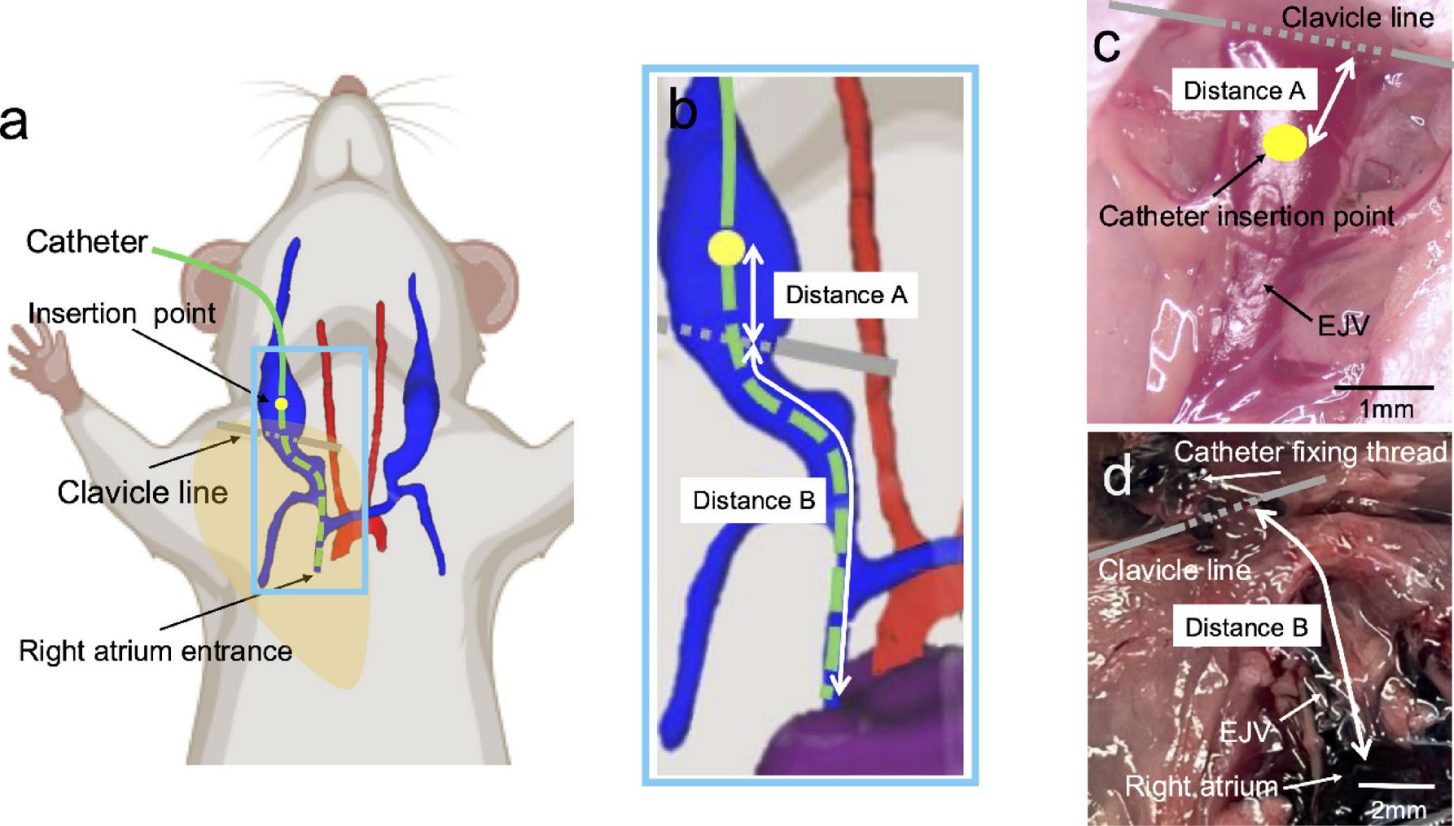
Schematic and image of catheter length. (**a**) An overall view shows catheter insertion through the external jugular vein. Blue indicates the external jugular and subclavian veins; red, the aortic arch and carotid artery; and purple, the heart. Green denotes the catheter; yellow indicates the point of insertion of the catheter into the external jugular vein. (**b**) Close-up view of Fig. 1a. Distance A is defined as the distance from the external jugular vein insertion point of the catheter to the clavicular line. Distance B is defined as the distance from the clavicular line to the superior vena cava-right atrial junction. (**c**) Full view of the external jugular vein in surgery. Distance A is determined intraoperatively. (**d**) Full view of the external jugular vein into which the catheter was inserted at autopsy. The sternum and ribs have been removed. Distance B was measured manually at autopsy. Distance A plus Distance B is the optimal catheter insertion length (catheter tip at the junction of the superior vena cava and right atrium) into the external jugular vein. EJV, external jugular vein.

**Fig. 4:**
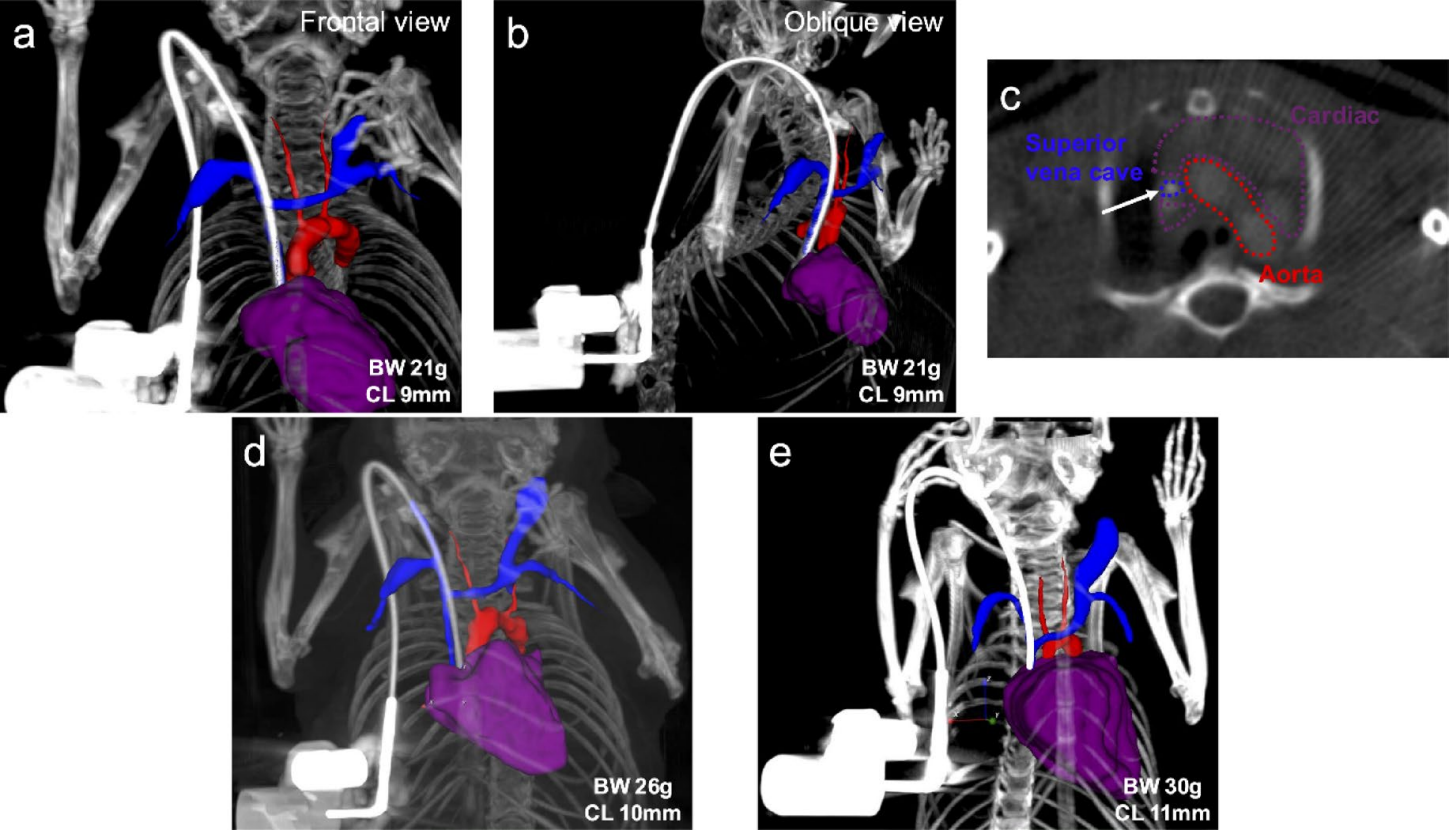
Comparison of different catheter insertion lengths in living mice. Imaging after catheter insertion obtained from an additional cohort of living mice (n = 5) allowed visualization and confirmation of optimal catheter tip location. Blue indicates the external jugular and subclavian veins; red, the aortic arch and carotid artery; and purple, the heart. A white high-intensity catheter filled with a contrast agent can be recognized as a curved material connected to the VAB. (**a**) 3D reconstruction demonstrating anterior view of a VAB with 9-mm catheter placement to the cardiovascular system of a 21 g mouse. (**b**) Oblique view of a 3D rendering of a 9 mm long VAB with catheter placement to the cardiovascular system of a 21 g mouse (**c**) Confirmation of the catheter tip on contrast-enhanced CT cross-sectional image. White arrow; the tip of the catheter located in the superior vena cava just before the right atrial entrance. (**d**) Anterior view of a 3D rendering of a 10 mm long VAB with catheter placement to the cardiovascular system of a 26 g mouse. (**e**) Anterior view of a 3D rendering of an 11 mm long VAB with a catheter implanted in a 30 g mouse. BW, body weight; CL, catheter length.

The optimal catheter insertion length was determined through a combination of intraoperative measurements and post-mortem examinations. Figure 3a,b show a schematic representation of the catheter insertion parameters used in this study. To ensure consistency, Distance A was defined as the distance from the clavicular line to the EJV catheter entry point (Fig. 3c). Distance B was defined as the distance from the clavicular line to the superior vena cava (SVC)-right atrial junction. This landmark where the SVC pierces the dome of the right atrium was deemed the optimal site for catheter implantation (Fig. 3d). The sum of Distances A and B represents the optimal catheter insertion length into the vein. Catheter insertion lengths obtained from intraoperative as well as autopsy findings and dependent on body weight are shown in Table 2. Distance A was nearly constant at 1.8 to 1.9 mm regardless of mouse weight. Distance B was 7.7 ± 0.5 mm for mice weighing 20 g to 25 g, 8.3 ± 0.7 mm for mice weighing 25 g to 30 g, and 9.3 ± 0.4 mm for mice weighing 30 g or more. To validate the predetermined catheter length specifications (Table 2), we performed imaging after catheter insertion in an additional cohort (n = 5) of live mice (Fig. 1), enabling visualization and confirmation of optimal catheter tip positioning. Figure 4 illustrates the catheter placement in a live mouse implanted with a VAB, visualized through a three-dimensional (3D) rendering of contrast-enhanced computed tomography (CT) images. The 3D vessel rendering was constructed from all directions and angles, and the frontal (Fig. 4a) and right oblique (Fig. 4b) images were critical elements in confirming the catheter tip position. The vein wall of the anterior half of the right EJV surrounding the catheter was removed during the rendering to better visualize the catheter’s trajectory and tip position. The position of the catheter tip was verified with a contrast-enhanced CT axial cross-sectional image (Fig. 4c). Following the guidelines in Table 2, VABs were implanted in mice weighing 21 g (Fig. 4a), 26 g (Fig. 4d), and 30 g (Fig. 4e) with catheters 9 mm, 10 mm, and 11 mm long, respectively. Catheter tip positioning, guided by weight-dependent length specifications, demonstrated that our standardized placement protocol is applicable to living mice and achieves the anatomical target location at the SVC-right atrial junction.

**Table 2:** Weight-Dependent Catheter Length Parameters.

Body weight	Distance A (mm)	Distance B (mm)	Optimal catheter length (A + B, mm)
20 g-25 g (n = 28)	1.78 ± 0.31	7.7 ± 0.5	9.5 ± 0.6
25 g-30 g (n = 23)	1.82 ± 0.28	8.3 ± 0.7	10.1 ± 0.8
30 g- (n = 9)	1.88 ± 0.22	9.3 ± 0.4	11.2 ± 0.5

Values are given as means ± standard deviation.

### Catheter patency

We evaluated catheter patency using a three-tier classification system: (i) complete patency—successful bidirectional flow, allowing both injection and blood aspiration via the VAB; (ii) partial patency—unidirectional flow, permitting injection but not blood aspiration via the VAB; and (iii) no patency—the absence of flow in either direction, precluding both injection and blood aspiration. Multiple factors potentially influencing catheter patency were systematically investigated. These included physical parameters such as catheter tip geometry, heparin concentration in the maintenance solution, and frequency of catheter maintenance procedures. The impact of these factors on maintaining 28-day patency was comparatively analyzed. Forty-six mice implanted with VABs were analyzed and stratified into four categories based on distinct combinations of factors influencing catheter patency (Fig. 1). Additionally, the study incorporated an evaluation of a 3 Fr catheter in an additional cohort (Fig. 1), which has a larger diameter than the 2 Fr catheters typically employed in murine models (Supplementary Fig. 1).

#### Square vs. round catheter tip

**Fig. 5:**
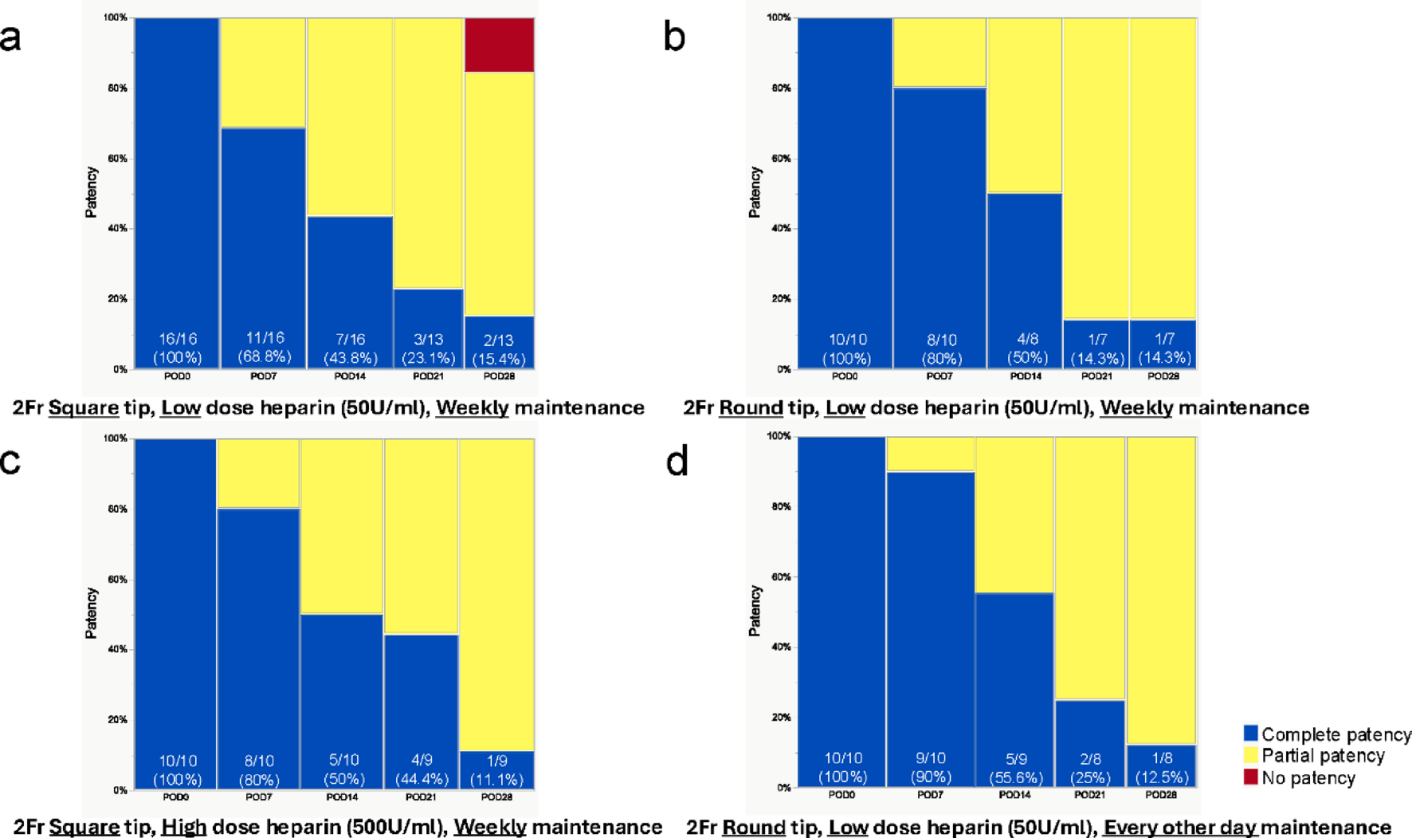
Catheter patency over time with different management methods. (**a**) The catheter tip geometry is square, heparinized saline used for flushing is a low dose (50U/ml), and catheter maintenance is weekly. (**b**) The catheter tip geometry is round, heparinized saline used for flushing is low dose (50U/ml), and catheter maintenance is weekly. (**c**) The catheter tip geometry is square, heparinized saline used for flushing is a high dose (500U/ml), and catheter maintenance is weekly. (**d**) The catheter tip geometry is round, heparinized saline used for flushing is low dose (50U/ml), and catheter maintenance is every other day. The numbers shown in the figure represent the percentage of complete patency.

First, we investigated the impact of catheter tip geometry (square *vs*. round) on vascular patency. The surgical technique and maintenance protocol were standardized across all subjects, with weekly administration of heparinized saline (50 U/mL) for catheter maintenance. The sole variable was the catheter tip geometry. Results are presented in Fig. 5a,b. We started the study with 16 mice in the square-tip group and 10 mice in the round-tip group; 3 mice at POD21 in the square-tip group, 2 mice at POD14, and 1 mouse at POD21 in the round-tip group were excluded from the analysis due to mortality. On POD7, complete patency was observed in 68.8% (11/16) of the square-tip group and 80% (8/10) of the round-tip group (*p* = 0.66, Fisher’s exact test). The incidence of partial patency increased over time in both groups. By POD28, complete patency rates had decreased to 15.4% (2/13) in the square-tip group and 14.3% (1/7) in the round-tip group (*p* = 0.53, Pearson’s chi-square test). Additionally, two cases of complete occlusion were observed in the square-tip group at POD28.

#### Low dose (50 U/ml) vs. high dose (500 U/ml) heparinized saline

Catheter tip geometry (square tip), surgical technique, and weekly maintenance frequency were standardized, and the dose of heparin used for flushing was compared between low (50 U/ml) and high (500 U/ml) concentrations. The results are shown in Fig. 5a,c. We started the study with 16 mice in the low-dose group and 10 mice in the high-dose group; 3 mice at POD21 in the low-dose group, and 1 mouse at POD21 in the high-dose group were excluded from the analysis due to mortality. At POD7, complete vascular patency was observed in 68.8% (11/16) of subjects in the low-dose group and 80% (8/10) in the high-dose group (*p* = 0.66, Fisher’s exact test). Both groups exhibited a progressive decrease in complete patency rates and a corresponding increase in partial patency rates over the postoperative period. Statistical analysis revealed no significant differences in patency outcomes between the low-dose and high-dose groups throughout the study duration (POD14: *p* = 0.66, Fisher’s exact test; POD21: *p* = 0.37, Fisher’s exact test; POD28: *p* = 0.42, Pearson’s chi-square test).

#### Comparison of weekly vs. every-other-day maintenance

We compared the effects of weekly and every other-day maintenance protocols on catheter patency. Catheter tip geometry (round tip), surgical technique, and heparinized saline concentration (50 U/mL) were standardized across all subjects. Results are presented in Fig. 5b,d. We started the study with 10 mice in the weekly maintenance group and 10 mice in the every-other-day group; 2 mice at POD 14 and 1 mouse at POD21 in the weekly maintenance group, and 1 mouse at POD14 and 1 mouse at POD21 in the every-other-day group were excluded from the analysis due to mortality. At POD7, complete vascular patency was observed in 80% (8/10) of subjects in the weekly maintenance group and 90% (9/10) in the every-other-day group (*p* = 1.00, Fisher’s exact test). Both groups exhibited a marked decline in complete patency rates, with approximately 50% reduction by POD14. This downward trend in complete patency continued throughout the subsequent postoperative period. Statistical analysis revealed no significant differences in patency outcomes between the two maintenance frequency groups at any observation time point (Comparison at all POD time points: *p* = 1.00, Fisher’s exact test).

#### 3Fr catheter with a square tip

**Fig. 6:**
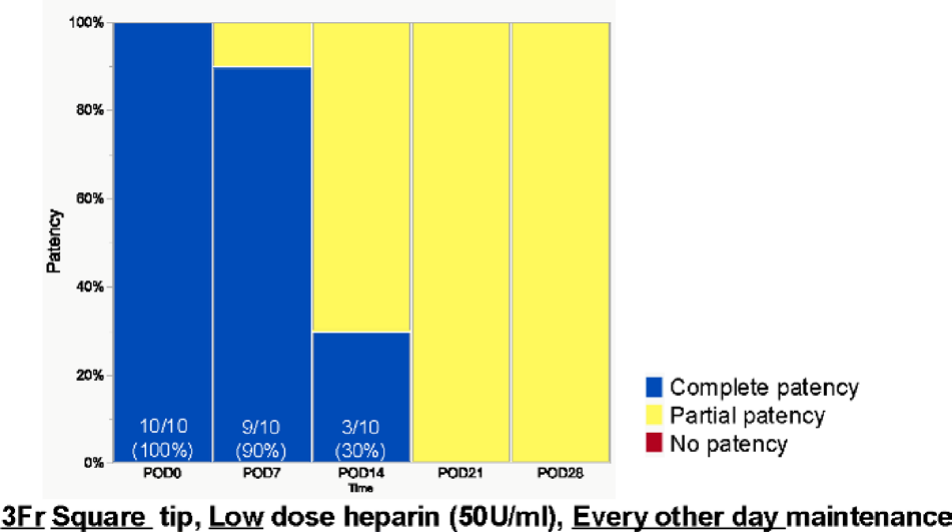
Catheter patency over time with 3Fr catheter. 3Fr diameter, the catheter tip geometry is square, heparinized saline used for flushing is low dose (50U/ml), and catheter maintenance is every other day. The numbers shown in the figure represent the percentage of complete patency.

Following unsuccessful attempts to improve partial patency rates through various experiments with 2 Fr catheters, including modifications to catheter tip geometry and maintenance protocols, we transitioned to larger 3 Fr catheters with square tips (Fig. 6). The internal and external diameters of the catheters were 0.43 × 0.69 mm for 2 Fr and 0.64 × 1.00 mm for 3 Fr, respectively. Surgical technique and maintenance protocol were standardized across all subjects, with catheter maintenance performed every other day using heparinized saline (50 U/mL). We conducted this analysis on 10 mice without mortality during the observation period. With the 3 Fr catheters, complete vascular patency was observed in 90% (9/10) of subjects at POD7, decreasing to 30% (3/10) at POD14, and 0% (0/10) at POD21. In all cases where complete patency was not maintained, partial patency was observed. Notably, the transition from complete to partial patency occurred earlier with 3 Fr catheters compared to the previous experiments with 2 Fr catheters.

### Biodistribution and clearance of the ICG with IV Injection via VAB

**Fig. 7:**
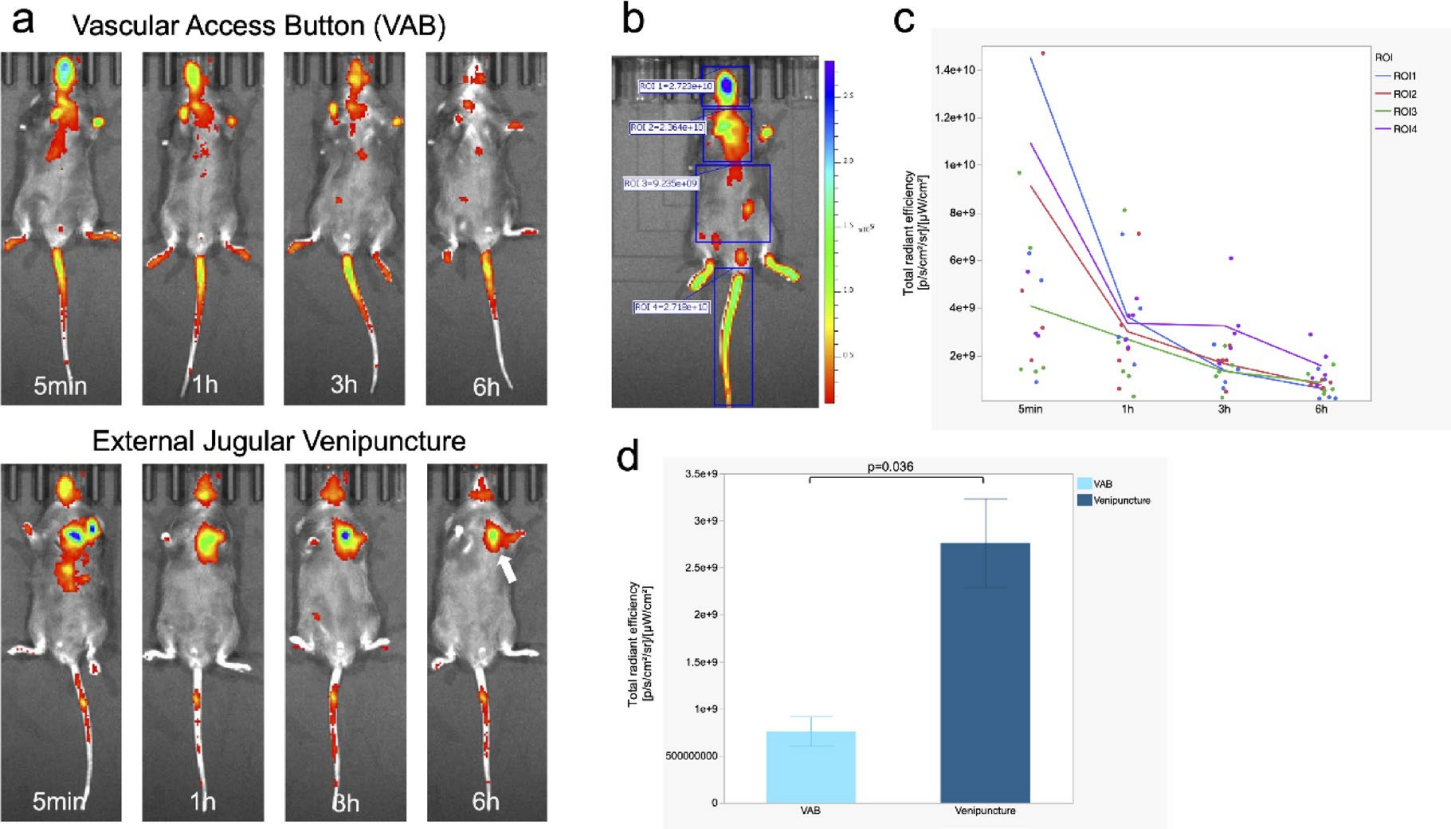
Biodistribution and clearance of the ICG with IV Injection via VAB. (**a**) Comparison of VAB vs. direct external jugular venipuncture over time. In both routes of administration, ICG is distributed throughout the body immediately after administration and is excreted in approximately six hours. Venipuncture mice still show relatively high ICG fluorescence at 6 h post-evaluation (White arrow). (**b**) The head, chest, abdomen, and tail were defined as regions of interest (ROI)1, ROI2, ROI3, and ROI4, respectively, and total radiant efficiency was calculated. (**c**) Trends over time in the total radiant efficiency of each ROI in mice administered ICG via VAB. The metabolic and excretory processes of ICG administered via VAB are represented by the quantitative decrease in fluorescence intensity in all ROIs. (**d**) Comparison of total radiant efficiency in ROI2 area at 6 h in VAB and venipuncture. Venipuncture mice have significantly higher fluorescence intensity (*p* = 0.036, Mann–Whitney U-test).

We compared the biodistribution and clearance of the indocyanine green (ICG) fluorescent dye with IV injection via VAB versus direct external jugular venipuncture, as visualized through an in vivo imaging system (IVIS) (Xenogen; Perkin Elmer, MA, USA). Five mice with IV injection via VAB and 3 mice with direct external jugular venipuncture were used for this analysis (Fig. 1). Figure 7a illustrates the temporal distribution of ICG as visualized by IVIS between these two administration routes. Figure 7b depicts a representative image of the regions of interest (ROI) defined in this study and the calculated total radiant efficiency. Figure 7c shows the total radiant efficiency over time at each ROI for mice administered ICG via VAB. Rapid systemic distribution of ICG was observed in both groups, with detectable fluorescence in the head and peripheral extremities at 5 min post-injection, indicating comparable biodistribution kinetics between VAB and direct venipuncture (Fig. 7a). ICG was gradually excreted into bile and cleared through the intestines. In VAB, the fluorescent signal is only weakly detectable 6 h after injection. The total radiant efficiency results in VAB corroborate these qualitative observations quantitatively (Fig. 7c). However, in mice receiving ICG via direct venipuncture, a relatively strong fluorescent signal persisted near the injection site (corresponding to ROI2) even 6 h post-injection (white arrow, Fig. 7a). A comparison of the total radiant efficiency in the ROI2 region at 6 h post-administration, as shown in Fig. 7d, revealed significantly higher values in mice that underwent direct external jugular venipuncture compared to those with VAB administration (*p* = 0.036, Mann–Whitney U-test).

## Discussion

This study represents the first comprehensive investigation into the technical feasibility and safety of implanting a VAB system in mice, along with practical strategies for maintaining catheter patency. Our findings demonstrate that VAB implantation can be achieved with high success rates and low postoperative mortality. The VAB system, utilizing a 2 Fr catheter, proves suitable for long-term, repeatable IV injections. However, study designs requiring concomitant blood collection may necessitate additional sampling routes, such as peripheral or central veins, independent of the VAB system. Furthermore, administering solutions via VAB offers potential advantages over direct venipuncture, particularly in terms of reliability and precision of intravascular dosing.

A comprehensive understanding of the EJV anatomy is crucial for overcoming challenges associated with catheter placement. Specifically, the procedure requires exposure of approximately 2 mm of the EJV and smooth insertion of a 2 Fr catheter, necessitating proficiency in both surgical and insertion techniques. The most challenging aspect of this procedure is catheter insertion, primarily due to the anatomical constraints of the EJV. The EJV traverses a narrow, short-diameter passage between the anterior clavicle and the pectoralis major muscle. Under microsurgical visualization, the EJV may appear to descend directly into the deep thoracic cavity. However, our anatomical observations reveal a crucial feature: the EJV follows a more complex path, initially ascending towards the body surface before descending through a constricted route between the pectoralis major muscle and the clavicle.

Previous studies have reported varying durations for EJV catheterization in mice, with total procedure times ranging from 20^15^, 40^16^, to 50^20^ min. Our findings demonstrate that the entire procedure, including catheter insertion, connection to the VAB, and subcutaneous implantation, can be completed more efficiently while maintaining a high technical success rate. Through accumulated experience since the initial introduction of the VAB procedure, we have significantly reduced operating times. This reduction in time not only enhances the feasibility of the technique but also potentially reduces stress on the animal subjects, which is crucial for subsequent experimental outcomes.

In our study, we observed a 28-day postoperative survival rate of 80.4%, indicating a relatively high level of procedural safety. Postmortem examinations of mice that experienced unexpected mortality revealed proper catheter placement within the EJV, but the direct cause of death remained unknown. The trend in A-MSS primarily reflected the postoperative recovery trajectory rather than serving as an early predictor of infectious complications. Our findings indicate that mice undergo a recovery period from the invasive effects of surgery lasting up to seven days postoperatively. Studies utilizing VABs should consider initiating experimental protocols after this seven-day recovery period to minimize confounding factors related to surgical stress and ensure more reliable results.

Determining and adjusting the optimal catheter tip placement intraoperatively presents significant challenges. Currently, this process relies primarily on the ease of catheter flushing and blood collection during the surgical procedure, which is correlated with the mouse’s body size as previously reported in the literature. Several studies have proposed guidelines for catheter insertion length. In female FVB mice, the length of the catheter tip to be inserted into the right atrium was defined as 11 mm at 25 g or less and 12 mm at 25 g or more^15^, 11.5 mm at 20 g, and 12.7 mm at 30 g^16^, and strain-specific standards have also been reported, though values are broadly consistent across strains^17^. However, these reports often lack clarity regarding the relationship between catheter insertion length, insertion site, and the intended final position of the catheter tip. To address this ambiguity, using the clavicle line as an anatomical landmark, we calculated the optimal insertion length to reach the entrance of the right atrium—considered the ideal position for a jugular vein catheter—for various bodyweight categories. The catheter insertion length obtained by the sum of these distances provides only a theoretically appropriate catheter insertion length. We validated this approach using contrast-enhanced CT imaging with 3D rendering, which supported the accuracy of our developed rule. Despite standardization efforts based on body weight, individual variations in skeletal structure and venous anatomy necessitate fine-tuning of catheter length during placement procedures. These adjustments are crucial for optimizing catheter function, particularly for facilitating efficient flushing and blood collection.

Maintaining the patency of indwelling IV catheters is key for optimal performance in long-term vascular access studies. In our 28-day postoperative observation period, complete catheter occlusion was a rare occurrence. However, we frequently encountered a condition we define as "partial patency," analogous to the clinical phenomenon often referred to as "one-way occlusion" or "partial occlusion”. This condition is characterized by the ability to flush the catheter but an inability to aspirate blood. Etiologically, partial occlusions can be categorized as either thrombotic or non-thrombotic. Thrombus formation is widely recognized as the primary mechanism underlying catheter occlusion^21,22^. This process involves the development of blood clots within the catheter lumen, around its external surface, or at the catheter tip^23^. Additionally, the activation of the coagulation cascade can lead to the formation of a fibrin sheath, further contributing to catheter obstruction^23,24^. On the other hand, non-thrombotic occlusion can result from various factors including catheter diameter, catheter material, catheter placement location, catheter tip geometry, maintenance protocols, catheter flushing frequency, as well as type and dose of flushing solution^25,26^. Our study focused on optimizing catheter management strategies to address modifiable non-thrombotic occlusive factors.

The selection of appropriate catheter diameter for EJV cannulation in mice presents a trade-off between ease of insertion and blood collection efficiency. Previous studies have demonstrated that 1.2 Fr catheters offer facile insertion but limited blood sampling capabilities, while 2 Fr catheters, despite more challenging insertion, allow for adequate blood collection^15^. Based on these considerations, our study protocol was initially designed to utilize 2 Fr catheters, prioritizing the ability to obtain sufficient blood samples for downstream analyses. This decision was made to optimize the balance between procedural difficulty and experimental utility. Also, regarding catheter material, polyurethane was selected for all procedures due to its superior characteristics, especially its high biocompatibility and flexibility. Our comparison of round and square 2Fr catheter tips in mice did not reveal significant differences in safety profiles or patency rates. Therefore, if economically feasible, round-tipped catheters may be preferable for long-term studies.

Although catheter maintenance protocols, such as frequency of maintenance and flushing solutions like heparin, are important in improving patency, we found no clear patency advantage in VAB with 2Fr catheters in mice. While all evaluated maintenance protocols adequately support long-term, repeated IV administrations, none prove satisfactory for consistent and reliable blood sampling via the VAB system. The frequency of catheter flushing varies, with reports recommending daily^15^, every other day^17,20^, or every 3 days^14^. Heparin doses in the maintenance protocols are also not firmly established in the literature and have varied widely, ranging from 10 U/ml^15,20^, 25 U/ml^14^, 100 U/ml^16^.

Contrary to expectations based on observations in larger animal models such as rabbits and ferrets, where 3 Fr catheters demonstrate extended patency for blood collection^10,11^, our study in mice revealed an unexpected outcome. The 3 Fr catheters exhibited a shorter duration of patency for blood sampling compared to the 2 Fr catheters. This finding contradicts the general trend observed in larger species and warrants further investigation into species-specific factors affecting catheter patency. These conflicting results may be attributed to the catheter-vessel ratio (CVR). CVR, defined as "the indwelling space or area consumed or occupied by an intravascular device inserted and positioned within a venous or arterial blood vessel", is essential in preventing catheter-associated thrombosis and occlusion^27^. The higher CVR of the 3 Fr catheter than the 2 Fr catheter may have contributed to an increased likelihood of vascular obstruction. While it is generally recommended that catheter diameter should not exceed one-third (33%) of the vessel’s inner diameter, applying this guideline in mice is challenging due to the small diameter of their superior vena cava. CT scanning with novel 3D rendering of the 2Fr catheter in vivo shows the catheter nearly completely occupying the superior vena cava. Three Fr catheters for cannulation of the EJV have been developed primarily for rats and larger animals, and their application in mice has not been reported. This absence in the literature may be attributed to the limited size of the central veins in mice and the anatomical challenges associated with the insertion of this relatively large size cannula. While we had successfully implanted VABs with 3 Fr catheters in mice, several factors had warranted careful consideration, including mouse body weight, the proximity of the EJV to the clavicle and pectoralis major muscle, and the dimensions of the superior vena cava. Although technically feasible, based on our experience, the implantation of 3 Fr catheters with VABs may be too challenging for routine vascular access in mice.

Since the implantation of 2 Fr catheters in mice is replete with issues regarding maintaining catheter patency, several catheter modifications could be beneficial. These include the addition of side holes in catheters, antimicrobial, hydrogel and hydrophilic coatings on catheters that reduce the risk of infection by preventing cell adhesion and biofilm formation, as well as heparin coating that reduces clot adhesion and provides beneficial antimicrobial activity^28–31^. The miniature dimensions of the 2 Fr catheter present significant bioengineering challenges for implementing modifications, necessitating advanced expertise in materials science and microfabrication techniques^32^.

The results of biodistribution and clearance of the ICG with IVIS demonstrated that the VAB system yields results comparable to conventional venipuncture. However, in mice administered ICG via conventional venipuncture, fluorescence was detectable at the injection site even 6 h post-administration, well beyond the expected excretion time of ICG. This persistent signal suggests potential extravasation of the injected solution and residual ICG along the needle track, even in cases where venipuncture appeared to be successful. These findings indicate that VAB administration may offer advantages over conventional venipuncture in studies requiring. These findings suggest that administration via VAB may be more beneficial than conventional venipuncture in studies requiring precise dosing, transplantation of cancer cells, or the administration of dermatoxic drugs.

There are several limitations to this study that should be considered. The most critical point to consider is that the technical success rate of 90.2% and 28-day survival rate of 80.4% we have presented may not be sufficient in terms of compliance with the 3Rs principle. Furthermore, the failure to maintain complete patency meant that the full benefits of VAB could not be maximized. From the perspective of ensuring animal welfare in experiments, improving these results in future research is essential. Second, our investigation was conducted using only C57BL/6 mice, which may limit the generalizability of our findings to other mouse strains. Third, the current study was limited by its relatively small sample size. Further studies may be needed, especially to determine if optimized catheter lengths can be used to obtain more precise and definitive anatomic locations in living mice. Additionally, we have not been able to identify a definite cause of death, including catheter-related bloodstream infection, or why obstruction affects patency, since the study focused on the practical management of the catheter and VAB. To address these limitations, a comprehensive evaluation may provide more robust data on the reproducibility, efficacy, and investigation of mechanisms of death and catheter occlusion.

This study demonstrates the feasibility, safety, and effectiveness of using a VAB in mice for long-term intravenous access. We established optimized protocols for catheter insertion length and maintenance, contributing to procedural reproducibility and improved research outcomes in murine models. Our findings support the broader application of VAB technology for studies requiring durable vascular access, facilitating a methodological framework for reliable preclinical research.

## Methods

This study is performed in accordance with relevant guidelines and regulations. All methods are reported in accordance with ARRIVE guidelines^33^.

### Animals

All experiments in this study were approved by the Institutional Animal Care and Use Committee of Johns Hopkins University (MO23M72). All male and female C57BL/6 mice were purchased from Charles River Laboratories (Wilmington, MA). All experiments were performed using adult C57BL/6 mice of both sexes. Mice were housed under a reversed 12:12 h, light: dark cycle and provided with food and water ad libitum.

### Surgical supplies

Polyurethane jugular vein catheter (2Fr) with square and round tips, 3Fr polyurethane jugular vein catheter with square tip, one channel vascular access button™ for mice, PinPort™ injectors, and protective aluminum cap for magnetic one channel mouse VAB were used (Instech Laboratories, Plymouth Meeting, PA). 5–0 and 4–0 silk surgical suture (MDSupplies, Davie, FL), dissecting microscope (AmScope, Irvine, CA)、general dissecting instruments (Roboz, Gaithersburg, MD)、chlorhexidine (MilliporeSigma, Rockville, MD), heparin solution (MilliporeSigma, Rockville, MD), carprofen (VetOne, Boise, ID), 21G and 18G needle (BD, Franklin Lakes, NJ) and 1 ml syringe (BD, Franklin Lakes, NJ) were used.

### Perioperative outcome

To evaluate the safety of VAB implantation in mice, we assessed surgical outcomes, including operative time**,** intraoperative external venous diameter, postoperative complications, and 28-day survival rate. A-MSS was developed as an indicator of infectious complications and is used to assess the postoperative recovery status of mice^19^. A-MSS is an improved scoring system based on the Murine Sepsis Score that incorporates additional quantitative measures and was developed to assess the severity of sepsis in mice^34^. It includes observational characteristics such as level of consciousness, activity, behavior, response to stimuli, respiratory rate, and quality of breathing, as well as other sensory indicators of blood sugar, body weight, and body temperature (Supplementary Table 1). The A-MSS is scored on a 0 to 40 point scale, with higher scores indicating worse condition of the mice. The A-MSS, which overlaps much with the postoperative recovery status assessment of mice, was evaluated in each mouse on postoperative days 1, 3, 7, and every 7 days postoperatively.

### Catheter insertion length optimization

Distance A was measured intraoperatively under general anesthesia; Distance A was the manually measured distance from the catheter insertion point to the clavicular line, selected based on EJV diameter, course, and estimated wall thickness. Euthanized mice were necropsied, the sternum and partial ribs were removed, and the thoracic cavity was freed. The fatty tissue of the mediastinum was removed, the EJV, superior vena cava and the cardiac were exposed, and Distance B was manually measured. Additionally, contrast-enhanced CT was used to assess catheter position, morphology, and insertion in live mice implanted with VABs. Mice implanted with our optimized weight-dependent catheter length VABs underwent CT imaging between 7 and 10 days postoperatively. Anesthesia was induced with 3% isoflurane and maintained at 1.5–2% during the evaluation. ExiTron™ nano 12000 (Viscover™, nanoPET Pharma GmbH, Berlin) was used as the contrast agent. All mice received 100 μL Viscover™ via the VAB, equivalent to 1200 mg iodine/kg body weight. CT scans were performed using a small animal PET/CT scanner (NanoPET/CT, Mediso Medical Imaging Systems, Budapest, Hungary). Vivoquant™ 2022 (Invicro, Boston, MA, USA) was utilized to create multi-planar reconstruction images and 3D renderings. Mice analyzed for catheter length optimization 28 days after catheter patency assessment and mice that underwent CT imaging were euthanized using CO2 inhalation.

### Catheter maintenance

Catheter maintenance, through flushing, is essential to maintain catheter patency, and catheter flushing is key. Typically, sterile saline, or heparinized saline, was used to ensure catheter patency. For catheter maintenance, mice were lightly anesthetized using an open-drop method with isoflurane. A syringe equipped with a Pinpoint injector was filled with heparinized saline and connected to the VAB. Initially, 50–100 μL of heparinized saline was injected to flush the catheter. Subsequently, blood aspiration was performed to confirm catheter patency. Following successful aspiration, a final flush of 50–100 μL heparinized saline was administered using the positive pressure technique. The access button was then sealed with its protective cap. The heparin concentration in the flush solution and the frequency of maintenance procedures adhered to the predetermined study protocol.

### Biodistribution and clearance of the ICG with IV Injection via VAB

ICG (MP biomedicals, OH, USA) was reconstituted in 1 ml of purified water. A dose of 0.5 mg/kg ICG was then diluted in phosphate-buffered saline to a final volume of 50 µl for administration via each route. ICG distribution in mice was assessed at 5 min, 1 h, 3 h, and 6 h postadministration using the IVIS system. Four ROIs were defined: head, chest, abdomen, and tail. The total radiant efficiency, a quantitative measure of fluorescence intensity specific to the IVIS system, was calculated for each ROI using Living Image software. This parameter allows for comparative analysis between samples. The IVIS system was configured with an excitation wavelength of 745 nm and an emission wavelength of 840 nm. These imaging parameters remained consistent across all subjects to ensure standardization and comparability of results.

### Statistical analysis

All statistical analyses were performed using SPSS software version 29 (IBM Corp, Armonk, NY, USA). Given the limited sample size and to enhance robustness against potential deviations from normality, we employed the Mann–Whitney U test to compare total radiant efficiency with IVIS. Statistical significance was set at *p* < 0.05. Fisher’s exact test and Pearson’s chi-square test were used to analyze categorical variables related to catheter patency for the postoperative course. To address the increased risk of Type I error due to multiple comparisons, the Bonferroni correction was applied for analyses involving repeated comparisons of catheter patency at multiple postoperative time points (POD7, 14, 21, 28). Specifically, the significance threshold (α) was divided by the number of comparisons (α = 0.05/4 = 0.0125). Only mutually exclusive data points were used for between-group comparisons, and each animal contributed a single, independent data point to any given statistical test.

## Supplementary Information

Below is the link to the electronic supplementary material.

Supplementary Material 1

Supplementary Material 2

## Data Availability

The data that support the findings of this study are available upon request from the corresponding authors.
