# Supplementary material for: Implantation of a vascular access button in mice
Source: Sci Rep. 2025 Oct 21;15:36627. doi: 10.1038/s41598-025-20542-4 (PMC12541028; doi:10.1038/s41598-025-20542-4)
Supplement: Supplementary file 1 — Supplementary Material 1 [file 41598_2025_20542_MOESM1_ESM.docx]

**Supplementary Fig. 1:** Differences in catheter tip geometry and diameter

**Supplementary Video 1.** Step-by-step VAB implantation technique

Anesthesia was induced by placing the mouse in a chamber filled with 3% isoflurane and 2 L/min O2. After induction of anesthesia, mice were fitted with an anesthetic mask covering the nasal region, and anesthesia was maintained with 1-2% isoflurane and 1 L/min O2 during surgery. The surgical field was shaved over the back where the access button would be implanted and over the right neck and chest where the catheter would be inserted. Both eyes were lubricated. The surgical field was disinfected three times with a 2% chlorhexidine solution. Carprofen (5 mg/kg) was administered subcutaneously for intraoperative and postoperative pain control. The mouse was placed prone on the operating table and a 15-mm skin incision was made on the back neck. The subcutaneous tissue and muscles were detached, and space was made under the right dorsal skin for an access button. A 7.5-mm hole was created at a site remote from the previous skin incision in order to guide the built-in port and magnetic pedestal from the subcutaneous area to the surface of the skin. The mouse was then placed supine and the extremities were fixed with tape. A 7-mm vertical incision was made in the skin in the cervical region above the clavicle, 5 mm lateral to the manubrium. All subsequent dissection was done utilizing microsurgical techniques and equipment. The fat and the membrane covering the blood vessels were separated, and the right EJV was skeletonized. The EJV was exposed from its superior aspect above the pectoralis major muscle to its junction with the internal jugular vein cranially. The EJV was ligated cranially, immediately proximal to its confluence with the internal jugular vein. Two loose ligatures were placed around the caudal portion of the EJV, adjacent to the pectoralis major muscle, to facilitate subsequent catheter fixation. A venotomy was performed on the EJV approximately 2 mm caudal to the pectoralis major muscle using a 21-gauge needle (18-gauge for 3 French catheter). The catheter was inserted and advanced to the predetermined optimal depth, as established by our previous measurements. Proper positioning was confirmed by successful blood aspiration and heparinized saline flush. The catheter was secured *in situ* using the previously placed ligatures. One ligature was specifically positioned to anchor the catheter, preventing dislodgement. The animal was then repositioned to a lateral recumbent posture, and a subcutaneous tunnel was created from the dorsal incision to the anterior right cervical region. The distal end of the catheter was guided through this tunnel to emerge at the dorsal site. Subsequently, the mouse was returned to a supine position. The catheter was trimmed to the appropriate length and connected to a VAB, which was implanted slightly right of the dorsal midline. Catheter patency was verified by successful blood aspiration and heparinized saline flush using a Pinpoint injector connected to the access button. The dorsal and cervical incisions were closed using 4-0 silk sutures.

**Supplementary Table 1.** Adapted Murine Sepsis Score (A-MSS)

| Score | 0 | 1 | 2 | 3 | 4 |
| --- | --- | --- | --- | --- | --- |
| Appearance | Coat is smooth | Patches of hair piloerected | Majority of back is piloerected | Piloerection may or may not be present; mouse appears “puffy” | Piloerection may or may not be present; mouse appears emaciated |
| Level of consciousness | Mouse is active | Mouse is active but avoids standing upright | Mouse activity is noticeably slowed. The mouse is still ambulant | Activity is impaired. Mouse only moves when provoked; movements have a tremor | Activity severely impaired. Remains stationary when provoked, with possible tremor |
| Activity | Normal amount of activity. Mouse is any of eating, drinking, climbing, running, and fighting | Slightly suppressed activity. Mouse is moving around bottom of cage | Suppressed activity. Mouse is stationary with occasional investigative movements | No activity | No activity. Mouse experiencing tremors, particularly in the hind legs |
| Response to stimulus | Mouse responds immediately to auditory stimulus or touch | Slow or no response to auditory stimulus; strong response to touch (moves to escape) | No response to auditory stimulus; moderate response to touch (moves a few steps) | No response to auditory stimulus; mild response to touch (no locomotion) | No response to auditory stimulus. Little or no response to touch. Cannot right itself if pushed over |
| Eyes | Open | Eyes not fully open, possibly with secretions | Eyes at least half closed, possibly with secretions | Eyes half closed or more, possibly with secretions | Eyes closed or milky |
| Respiration rate | Normal, rapid mouse respiration | Slightly decreased respiration (rate not quantifiable by the eye) | Moderately reduced respiration (rate at the upper range of quantifying by the eye) | Severely reduced respiration (rate easily countable by the eye, 0.5 s between breaths) | Extremely reduced respiration (>1 s between breaths) |
| Respiration quality | Normal | Brief periods of laboured breathing | Laboured, no gasping | Laboured with intermittent gasps | Gasping |
| Rectal temperature (°C) | 36-38 | > 38 | < 36- ≥ 35 | < 35- ≥ 34 | < 34 |
| Glycemia (mg/dL) | ≥148 | ≤148-≥122 | <122-≥58 | <58->40 | ≤ 40 |
| Relative body weight loss (%) | 0-5 | 5-10 | 10-15 | 15-20 | > 20 |
